# Supervision training in healthcare: a realist synthesis

**DOI:** 10.1007/s10459-019-09937-x

**Published:** 2019-11-05

**Authors:** Charlotte E. Rees, Sarah L. Lee, Eve Huang, Charlotte Denniston, Vicki Edouard, Kirsty Pope, Keith Sutton, Susan Waller, Bernadette Ward, Claire Palermo

**Affiliations:** 1 Monash Centre for Scholarship in Health Education (MCSHE), Monash University, 27 Rainforest Walk (Building 15), Clayton Campus, Clayton, VIC 3800 Australia; 2 Department of Medical Education, Melbourne Medical School, The University of Melbourne, Melbourne, VIC Australia; 3 Department of Occupational Therapy, Faculty of Medicine, Nursing and Health Sciences, Monash University, Frankston, VIC Australia; 4 Monash Rural Health, Faculty of Medicine, Nursing and Health Sciences, Monash University, Warragul, VIC Australia; 5 Monash Rural Health, Faculty of Medicine, Nursing and Health Sciences, Monash University, Bendigo, VIC Australia

**Keywords:** Supervision, Training, Health, Realist synthesis

## Abstract

Supervision matters: it serves educational, supportive and management functions. Despite a plethora of evidence on the effectiveness of supervision, scant evidence for the impact of supervision training exists. While three previous literature reviews have begun to examine the effectiveness of supervision training, they fail to explore the extent to which supervision training works, for whom, and why. We adopted a realist approach to answer the question: to what extent do supervision training interventions work (or not), for whom and in what circumstances, and why? We conducted a team-based realist synthesis of the supervision training literature focusing on Pawson’s five stages: (1) clarifying the scope; (2) determining the search strategy; (3) study selection; (4) data extraction; and (5) data synthesis. We extracted contexts (C), mechanisms (M) and outcomes (O) and CMO configurations from 29 outputs including short (n = 19) and extended-duration (n = 10) supervision training interventions. Irrespective of duration, interventions including mixed pedagogies involving active and/or experiential learning, social learning and protected time served as mechanisms triggering multiple positive supervisor outcomes. Short-duration interventions also led to positive outcomes through mechanisms such as supervisor characteristics, whereas facilitator characteristics was a key mechanism triggering positive and negative outcomes for extended-duration interventions. Disciplinary and organisational contexts were not especially influential. While our realist synthesis builds on previous non-realist literature reviews, our findings extend previous work considerably. Our realist synthesis presents a broader array of outcomes and mechanisms than have been previously identified, and provides novel insights into the causal pathways in which short and extended-duration supervision training interventions produce their effects. Future realist evaluation should explore further any differences between short and extended-duration interventions. Educators are encouraged to prioritize mixed pedagogies, social learning and protected time to maximize the positive supervisor outcomes from training.

## Introduction

Supervision matters in health and human services. While definitions of supervision vary across the literature (Martin et al. 2017), Proctor’s popular model outlines three purposes of supervision: facilitating consistent and quality practice in supervisees (managerial function), helping the development of supervisees’ knowledge, skills, attitudes and practices (educational function), plus providing supervisees with support and validation (restorative function) (Proctor 1987; Brunero and Stein-Parbury 2008; Dilworth et al. 2013; Gonsalvez and Milne 2010). See Box 1 for varying examples of definitions for supervision. An effective supervisor skilfully provides feedback, teaches, fosters collaborative learning, understands the expectation of their supervisees and is organized (Gibson et al. 2018). While supervision training is thought to enhance supervision effectiveness (Martin et al. 2014; Fitzpatrick et al. 2012; Dilworth et al. 2013; Chu et al. 2016), supervisors in health and human services consistently lack such training (MacDonald 2002; Spence et al. 2001; Hoge et al. 2011; Butterworth et al. 2008). Despite a plethora of evidence on the quality and effectiveness of supervision over the last 20 years (Spence et al. 2001; Hill et al. 2014; Newton et al. 2016), scant evidence evaluating the impact of supervision training exists. Only three reviews exist in the literature focusing on supervision training (Milne et al. 2011; Gonsalvez and Milne 2010; Tsutsumi 2011). While these reviews begin to offer insights into the effectiveness of supervision training, they largely focus on the positive outcomes of supervision training without *explicitly* discussing the complexities around how training interventions influence the quality or effectiveness of supervision. To address this gap in the supervision training literature, we conducted a realist synthesis to explore the extent to which supervision training works (or does not work), for whom and under what circumstances, how and why.

**Box 1:** Example definitions of supervision

“Clinical supervision is a process of professional support and learning in which nurses are assisted in developing their practice through regular discussion time with experienced and knowledgeable colleagues…” (Brunero and Stein-Parbury 2008, p. 87) “Supervision is any activity where more experienced health professionals provide less experienced health professionals with opportunities that enable these health professionals to achieve learning, to receive support, and to improve the quality and safety of their practice” (Fitzpatrick et al. 2012, p. 462) “Supervision is a forum where supervisees review and reflect on their work in order to do better. Practitioners bring their actual work-practice to another person (individual supervision), or to a group (small group or team supervision), and with their help review what happened in their practice in order to learn from that experience” (Caroll 2007, p. 36) “The formal provision, by approved supervisors, of a relationship-based education and training that is work-focused and which manages, supports, develops and evaluates the work of colleagues…” (Milne 2007, p. 439) “The term clinical supervision is defined as a formal process of professional support and learning which enables individual practitioners to develop knowledge and competence, and is acknowledged to be a life-long process…” (Martin et al. 2014, p. 201)	

### Diversity and complexity of supervision training interventions

While many argue for the importance of training to enhance supervision effectiveness (Kilminster and Jolly 2000), supervisors often carry out their supervision roles without any specific training (Hoge et al. 2011). For supervisors who *do* experience supervision training, they can experience a wide diversity in training with respect to content, mode, pedagogical strategies and duration. Supervision training content often focuses on the development of supervisor knowledge (e.g. definitions, models, methods, responsibilities, legal/ethical aspects) (Kilminster and Jolly 2000; Spence et al. 2001; Newton et al. 2016), and/or skills (teaching, assessment, feedback, counselling, leadership, interpersonal) (Kilminster and Jolly 2000; Hill et al. 2014; McKellar and Graham 2017). Supervision training modes include face-to-face, online or blended approaches. Pedagogical strategies also vary including didactic (e.g. presentations, videotaped demonstrations), active (e.g. small group discussions) and/or experiential learning (e.g. role-play, feedback) (Spence et al. 2001; Hoge et al. 2011; Pollock et al. 2017). The duration of supervision training ranges from one-off, short-term interventions (such as a 2-day workshop) to extended-duration interventions over many months that are punctuated by mini-interventions such as monthly supervision sessions (Spence et al. 2001). Although competency frameworks for supervision have been developed to guide supervision training (Health Workforce Australia 2014), some scholars argue that a lack of specificity still exists in terms of what and how supervision training should be conducted (Reiser and Milne 2014; Alfonsson et al. 2018). Furthermore, supervision training interventions have been criticised for lacking theoretical and evidence-based foundations (Kilminster and Jolly 2000). Therefore, we designed this realist synthesis to address these criticisms and gaps in the current literature.

### A realist approach to supervision training

Given the diversity and complexity of supervision training interventions, a realist synthesis was used to better understand how and why supervision training interventions produce their effects. A realist approach is theory-driven, so facilitates the development and modification of program theories accounting for how and why interventions work (or do not work) and for whom and when (Wong et al. 2012, 2016). This approach is underpinned by scientific realism, which asserts that it is not interventions that create change; rather, it is *people* who create change (Pawson and Tilley 1997). Interventions are thought to lead to outcomes through the operation of mechanisms, that is, the resources proffered by an intervention and the ways in which this influences participants’ reasoning (Dalkin et al. 2015). Furthermore, there is an appreciation that this complex relationship is context-dependent (Sholl et al. 2017; Ajjawi et al. 2018). Outcomes of any intervention can be affected by the range of conditions within any given setting, which are often sociocultural (Jolly and Jolly 2014). The basic premise is: what works for one person might not work for another; and what works in one circumstance might fail to work in another (Wong et al. 2016). While the context–mechanism–outcome (CMO) relationship is not necessarily straightforward or linear, contextual aspects are thought to trigger particular mechanisms in response to an intervention leading to particular outcomes (Jolly and Jolly 2014). See Box 2 for a glossary of key realist terms.

**Box 2:** Glossary of realist terms

Contexts can be described as: “the conditions that an intervention operates in (often but not exclusively sociocultural)” (Taylor et al. 2007, p. 28). Context can refer to individuals participating in programs, stakeholder interrelationships, institutional arrangements in which programs sit and/or wider cultural, economic and/or societal settings for programs (Pawson 2018). Mechanisms can be described as: “underlying entities, processes or structures which operate in particular contexts to generate outcomes of interest” (Astbury and Leeuw 2010, p. 368). Mechanisms are typically hidden, sensitive to contextual variations and generative of outcomes (Astbury and Leeuw 2010). Outcomes can be described as the desired products of a program and/or the program’s observed products (Yardley et al. 2015; Jolly and Jolly 2014). Context–mechanism–outcome configurations (CMOCs) can be described as heuristics employed “by some realists during analysis to identify the causal links between context, mechanism and outcomes” (Marchal et al. 2018, p. 83). Demi-regularities can be described as: “prominent recurrent patterns of contexts and outcomes… in the data” (Wong et al. 2013, p. 9). Program Theory can be described as: “a plausible and sensible model of how a program is supposed to work” (Bickman 1987, p. 5). A program theory therefore is an explanatory account of how a program works, under what circumstances and for whom (Astbury and Leeuw 2010). Such a theory-driven approach should include both the development of, and testing and refinement of, program theory (Astbury and Leeuw 2010). Middle-range theory (MRT) can be described as theory situated: “between the minor but necessary working hypothesis… and the all-inclusive systematic efforts to develop a unified theory that will explain all the observed uniformities of social behavior, social organization and social change” (Merton 1968, p. 83). MRT can be considered formal theory providing a bridge to existing knowledge about a topic (Marchal et al. 2018).	

### Developing an initial program theory from non-realist supervision training reviews

**Fig. 1:**
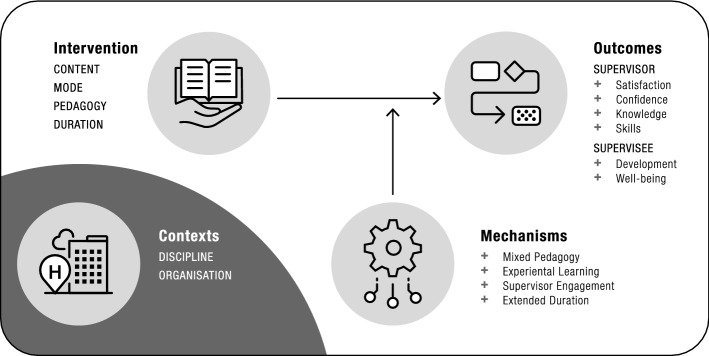
Initial program theory

Three supervision training reviews, one narrative and two systematic, have so far been published in the literature (Milne et al. 2011; Gonsalvez and Milne 2010; Tsutsumi 2011). While none of these employed realist approaches, nor did they include middle-range theory (MRT) specific to education apart from mentioning Kolb’s (1984) experiential learning (Milne et al. 2011), we applied realist logic in our reading of these reviews to develop an initial program theory (IPT: Fig. 1). We developed this IPT based on our identification of contexts, mechanisms, outcomes and context–mechanism–outcome configurations (CMOCs) for the supervision training interventions across the three papers. Firstly, studies included in these three reviews had several different contexts including health and human services (e.g. psychology, mental health, allied health), plus commercial contexts such as sales and insurance (Milne et al. 2011; Gonsalvez and Milne 2010; Tsutsumi 2011). Secondly, the supervision training interventions outlined within these three reviews were complex and diverse in terms of the: (a) content such as knowledge, skills and attitudes; (b) mode of delivery including face-to-face and online learning; (c) pedagogical strategies employed including theoretical and experiential learning; and (d) duration of the interventions including short (e.g. half-day) and extended durations (e.g. year) (Milne et al. 2011; Gonsalvez and Milne 2010; Tsutsumi 2011). Third, a variety of (mostly) positive outcomes were identified within the three reviews and related to supervisors (e.g. improved satisfaction, confidence, knowledge, skills) and supervisees (e.g. improved satisfaction and mental health). Fourth, we were also able to identify some mechanisms in the reviews to explain why interventions produced their effects e.g. supervision training interventions having an appropriate balance between didactic and experiential learning methods and extended durations enhancing engagement. Finally, we were able to identify two distinct CMOCs in two of the reviews:

- Within mental health supervision [C] clinical supervision training [I] leads to supervisor and supervisee development [O] through having a blend of pedagogic methods such as feedback, educational role-play and modelling [M] (Milne et al. 2011).
- Within the workplace [C], supervision training [I] leads to improved supervisor knowledge and behaviour, plus enhanced supervisee mental health [O] through improved knowledge and behavioural modification [M] (Tsutsumi 2011).

### Study aim and research questions

Although we have been able to identify contexts, mechanisms and outcomes and two CMOCs for supervision training interventions from previous reviews using realist logic, and develop an IPT, this current realist synthesis aimed to extend current literature reviews to develop a modified program theory (MPT). It aimed to review the published supervision training literature within health and human services to answer the novel research question: To what extent do supervision training interventions work (or not), for whom and in what circumstances, and why?

## Methods

The review protocol registered on PROSPERO (CRD42018094186) and published (Lee et al. 2019) was underpinned by Pawson’s five stages of realist review: (1) clarifying scope; (2) searching for evidence; (3) study selection; (4) data extraction; and (5) data synthesis (Pawson et al. 2005). Although presented in a linear fashion, stages were conducted iteratively with some overlap. The review methods and findings follow the RAMESES publication standards for realist syntheses (Wong et al. 2013).

### Clarifying the scope

A matrix identifying existing primary literature/empirical studies, literature reviews, search terms and their synonyms was created, generating numerous search terms. With the help of a medical librarian (see acknowledgements), pilot searches were conducted using several databases to test search terms (those identified as keywords in other published supervision training outputs, plus synonyms familiar to our multidisciplinary team), Boolean operators and proximity searching. Note that our original scope for this realist synthesis was broad including health (e.g. medicine, nursing, allied health etc.) *and* human services (e.g. housing, disability, children services, youth and family services, alcohol and drug services, out of home care etc.), consistent with our funding (Victorian Department of Health and Human Services). Furthermore, our scope was also broad in terms of interventions (e.g. workshops, online education, lectures), professions (e.g. nursing, physiotherapy, pharmacology, mental health), contexts (e.g. hospitals, universities, training centers, community services) and levels of learner (e.g. undergraduate students, postgraduate trainees, peers and colleagues).

### Searching for empirical evidence

A final and comprehensive search of the literature was conducted in May 2018 by SLL, with input from the medical librarian and co-authors. Note that none of these final searches were limited by date. Key search terms and phrases included supervisor terms (e.g. supervisor, practice educator, clinical educator, preceptor) and training terms only (e.g. education, professional development, train-the-trainer). Given the breadth of our search, we did not include search terms relating to interventions, professions, contexts or levels of learner, as advised by our medical librarian. For a full list of search terms see the protocol (Lee et al. 2019). Key terms were combined with proximity searching, Boolean operators, truncations and asterisks. Furthermore, searches were adapted to meet the operative requirements of each database including: Educational Resources Information Center (ERIC, ProQuest); Australian Public Affairs Information Service (APAIS, Informit); Social Services Abstracts (ProQuest); Scopus; PsycINFO (Ovid); MEDLINE (Ovid); and Cumulative Index to Nursing and Allied Health Literature (CINAHL Plus, Ebsco). An example of a CINAHL search strategy is included in Box 3. Citations and reference lists of included studies were hand searched to identify additional relevant studies.

**Box 3:** Search strategy example of CINAHL search

(supervisor* OR mentors OR mentor OR mentoring OR instructor* OR “placement educator*” OR “practice educator*” OR trainer* OR preceptor OR preceptors OR “clinical teacher*” OR “clinical educator*” or “fieldwork educator*”) N2 (training* OR education OR educating OR workshop*) Supervision N1 (training OR education OR educating OR workshop*) “train the trainer*” (“professional development” OR “faculty development” OR “personal development” OR CPD) N2 (supervisor* OR mentors OR mentor OR mentoring OR instructor* OR “placement educator*” OR “practice educator*” OR trainer* OR preceptor OR preceptors OR “clinical teacher*” OR “clinical educator*” OR “fieldwork educator*”)	

**Fig. 2:**
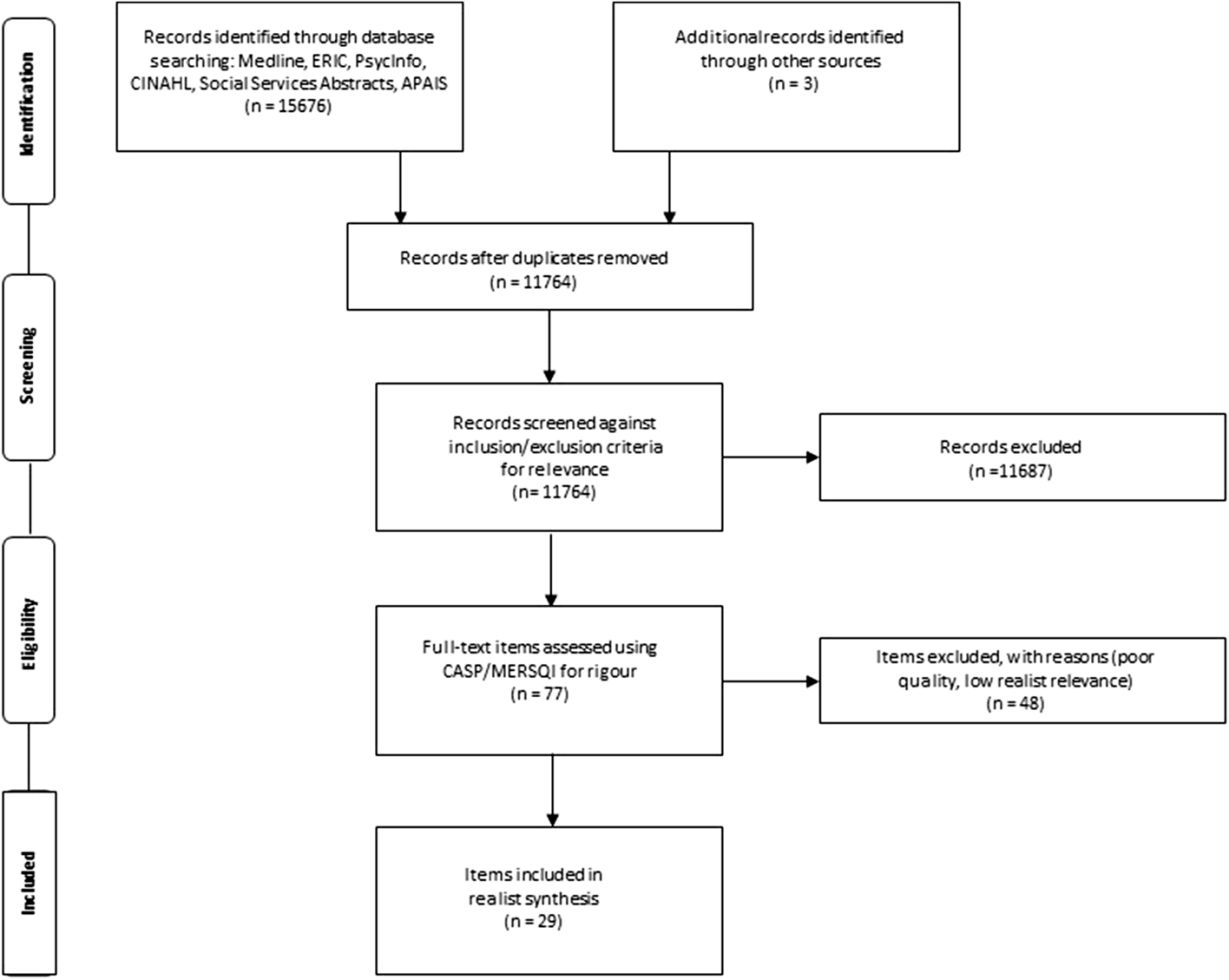
PRISMA

The first search elicited 15,676 outputs across all databases. Once duplicate results were removed, 11,764 outputs remained. All outputs were exported to Covidence software (© Covidence 2019) for management. The searching and selection process is summarised in the PRISMA diagram (see Fig. 2). Inclusion and exclusion criteria are shown in Table 1. Given the number of outputs identified, non-peer-reviewed outputs were excluded.

**Table 1:** Inclusion and exclusion criteria

Inclusion criteria	Exclusion criteria
Relate to one or more of the research questions	Literature relating to research supervision
Relevant population: literature must relate to either health/healthcare, housing, children services, youth and family services and/or mental health disciplines within the health and human service workforces	
Relevant intervention: literature must relate to supervision training interventions	
Literature must be written in the English language	
Peer-reviewed outputs only	
Primary/empirical research only	

### Study selection and appraisal

All authors (except EH) conducted initial assessments of outputs’ relevance using Covidence. Each analyst first participated in a calibration exercise of ten titles and abstracts using the inclusion criteria, with subsequent team-based discussions, before analyzing their own set of titles and abstracts. Each author (except EH) then screened a roughly equal portion of the titles and abstracts of studies retrieved using the search strategy (and those retrieved from hand searched references) against the inclusion criteria (see Table 1). Any ambiguities at this stage (i.e. outputs selected as ‘maybe’ in Covidence) were checked by a second independent researcher and resolved through discussion (SLL). Five percent of the 11,764 outputs examined for relevance was therefore double-checked at this stage (SLL) (Brennan et al. 2014).

Following this initial assessment of relevant titles and abstracts, 77 outputs remained. The full text of these outputs were retrieved and all authors assessed a roughly equal portion of outputs for rigour, after first participating in a second calibration exercise involving two full-text outputs, with consequent team-based discussions. Rigour was determined to understand whether the methods used to generate data were credible and trustworthy (Abrams et al. 2018). All authors checked rigour using either the Critical Appraisal Skills Programme (CASP) qualitative checklist (for qualitative or mixed methods studies) (Critical Appraisal Skills Programme 2018), or the Medical Education Research Study Quality Instrument (MERSQI) (for quantitative studies) (Cook and Reed 2015; Reed et al. 2009). At the same time as assessing rigour, we also reexamined relevance based on the full-text outputs, a process which we termed ‘realist relevance’. This meant that the outputs were judged in terms of whether they could contribute to the development of our IPT (Wong et al. 2013; Abrams et al. 2018). Assessment of ‘realist relevance’ was based on a 0–3 scale where 0 = the article lacked richness to enable the identification of contexts (C), mechanisms (M), outcomes (O) or context–mechanism–outcome configurations (CMOCs) and could therefore not help in the development of our IPT. At the other end of the scale, a paper received 3 = the article was sufficiently rich to identify CMOCs and could help develop our program theory. Finally, each paper was given an overall judgement (include, exclude, borderline) for rigour and realist relevance combined. Any outputs assessed as borderline for rigour and relevance (approximately 57%) were checked by a second author and any disagreements resolved through discussion (SLL and EH) (Brennan et al. 2014). The final sample of included outputs was 29.

### Data extraction

All authors (except VE and BW) extracted data after a third calibration exercise involving analysts’ extraction of two full-text outputs, with subsequent team-based discussions. The data extraction of the 29 outputs included: study characteristics (e.g. publication year, study methodology); contexts (e.g. study setting, profession, level of supervisor experience, country); intervention characteristics (e.g. content, mode, pedagogical strategies, duration); types of participants (e.g. clinical teachers); mechanisms and outcomes (note that outcomes and/or mechanisms could be positive or negative and pertain to supervisors or supervisees); CMOCs; and MRT. Contexts (C), Mechanisms (M) and Outcomes (O) and CMOCs for each supervision training intervention were highlighted on the 29 outputs and notes added by the data extractors. These highlights and notes were then transferred to tables using Microsoft Word (Microsoft, Windows 10) collating C, M, and Os and CMOCs both within and across our final sample. Note that during this process we labelled outcomes and mechanisms underpinning those outcomes as either positive (+) or negative (-). Inspired by other realist syntheses (Abrams et al. 2018), in order to elicit this information we made interpretations of meaning (e.g. does the relevant text provide sufficient data that could be interpreted as operating as contexts, mechanisms and/or outcomes?). Seven outputs (24%) were double-checked by a second extractor (mostly EH) at this data extraction stage, with any discrepancies resolved through discussion (SLL).

### Data synthesis

To synthesise the large amount of extracted data, we first divided our data into two categories based on the duration of interventions (either short or extended durations), given that intervention duration was flagged as an important intervention component *and* a mechanism underpinning positive outcomes in our IPT (Fig. 1). Note that *short* durations were defined as one-off interventions or interventions with multiple sessions but within a restricted time period (e.g. less than 1 week). Conversely, interventions with *extended* durations were defined as those conducted over many months (and sometimes years), with extended time periods between multiple sessions (e.g. monthly). Microsoft word tables including CMOCs with supporting illustrative quotes from the outputs were used for this data synthesis stage. Led by CER, four authors (CER, SLL, EH and CP) examined the data in these tables to identify demi-regularities (i.e. recurrent CMOCs) (Lee et al. 2019) across the 29 outputs with 139 original CMOCs identified across interventions with short (87 CMOCs) and extended durations (52 CMOCs). Inspired by other realist syntheses (Abrams et al. 2018), we asked questions like: is this CMOC found elsewhere in the same or other documents? How does this CMOC interplay with our IPT? How might this CMOC develop our program theory? Note that at this stage, CMOCs that were considered tangential to these demi-regularities or did not contribute to our MPT were removed from the final tables presented in this paper, leaving 74 final CMOCs (with 42 CMOCs for short-duration interventions, and 32 CMOCs for extended-duration interventions).

## Results

Following the assessment of rigour and realist relevance, 29 outputs remained in the final synthesis based on 28 studies; one study being presented across two outputs (Sandau et al. 2011; Sandau and Halm 2011). The final sample of outputs consisted of eight qualitative, eleven quantitative and ten mixed methods studies. Studies were conducted in various countries including the USA (n = 10), Australia (n = 5), UK (n = 3), Jordan (n = 2), Sweden (n = 3), Canada (n = 2), Netherlands (n = 1), Taiwan (n = 1) and Pakistan (n = 1), with one paper conducted across multiple counties (Myrick et al. 2011). Study interventions included face-to-face only (n = 24), online only (n = 4) and blended approaches including face-to-face *and* online components (n = 1). Interventions were either of short (n = 19) or extended durations (n = 10). There was a vast array of disciplines involved in the final sample including nursing (n = 9), medicine (n = 2) and allied health professions (n = 14), with some outputs including multiple disciplines (n = 4) (e.g. Carlson and Bengtsson 2015). In keeping with our IPT, data extraction and synthesis is presented separately for short (Table 2) and extended-duration interventions (Table 3).

**Table 2:** Data extraction for short-duration interventions

References	Study methods	Intervention	Settings	Middle-range theories
Al-Hussami et al. (2011)	Quantitative Experimental 68 registered nurses (RNs) randomly assigned to experimental (n = 30) or control group (n = 38) Objective assessment of their precepting knowledge	Knowledge and skills F2F PS: didactic and active learning 1 week with 4-h sessions (number of sessions and time between sessions unknown)	Nursing Clinical instructors Hospitals Jordan	King’s theory of goal attainment (King 1997)
Busari et al. (2006)	Quantitative Quasi-experimental 27 medical resident preceptors assigned to an experimental (n = 14) or control group (n = 13) Self-perception of the workshop and supervisees’ assessment of preceptors’ teaching	Knowledge and skills F2F PS: not disclosed 2 days	Medicine: paediatrics, and obstetrics and gynaecology Preceptors Teaching hospitals Netherlands	None
Carlson and Bengtsson (2015)	Qualitative Interpretive 27 focus group participants Self-perception of the intervention and self-assessment of learning outcomes	Knowledge and skills F2F PS: didactic, active and EL 40 h	Multiple disciplines: nursing, occupational therapy and biomedical science Clinical preceptors University Southern Sweden	Adult learning theories (not specified)
Clipper and Cherry (2015)	Quantitative Quasi-experimental 18 trained and 41 untrained nursing preceptors Supervisees’ assessment of preceptors’ teaching and self-assessment of their own student-to-nurse transition experience	Knowledge and skills Blended (5 online modules and a F2F course) PS: didactic, active and EL 3-h online modules and 1-day course	Nursing Preceptors Acute care hospitals USA	Boychuk Duchscher’s theory of transition shock (Boychuk Duchscher 2009)
Cox et al. (2017)	Quantitative Experimental, longitudinal 187/202 pharmacists completed the evaluation Self-assessment of learning outcomes	Knowledge Online PS: didactic 5–8 min in each video episode (12 episodes in total)	Pharmacology Adjunct and full-time faculty preceptors, and novice preceptors Colleges USA	Adult learning principles (not specified)
Eckstrom et al. (2006)	Qualitative Quasi-experimental, longitudinal pre/post-test 24 participants (experimental) and 44 (control group) Supervisees’ assessment of precepting quality and supervisors’ self-assessment of learning outcomes	Knowledge and skills F2F PS: didactic and EL half-day	Internal medicine Ambulatory preceptors University hospital, Veterans hospitals, and community sites USA	None
Ford et al. (2013)	Mixed methods Pre- and post-test 93 nurses and midwives Self-assessment of learning outcomes and perceptions of being preceptors	Skills F2F PS: active and EL 1 day	Nursing and midwifery Junior and senior preceptors A 400-bed tertiary referral hospital Australia	Experiential learning (not specified) Reflective practice (not specified)
Gillieatt et al. (2014)	Mixed methods Pre- and post-intervention survey 90/94 participants completed the pre and post surveys Self-assessment of learning outcomes	Knowledge and skills F2F PS: didactic and active learning 1 day	Multiple professions: medicine, nursing and allied health Experienced and novice preceptors Government and private organisations Australia	None
Henderson et al. (2006)	Qualitative Longitudinal 36 registered nurses Subjective assessment of learning outcomes	Knowledge and skills F2F PS: didactic, active and EL 2 days	Nursing Novice preceptors Acute tertiary referral center Australia	None
Hook and Lawson-Porter (2003)	Qualitative Triangulation 22 allied health professionals Perceptions of the workshop and self-assessment of learning outcomes	Knowledge and skills F2F PS: didactic and active learning 3 days	Allied health Novice preceptors Clinical setting UK	None
Lee et al. (2017)	Qualitative Experimental 13 nurse preceptors (NPs) and 11 new graduate nurses (NGNs) Objective assessment of learning outcomes, self-assessment and supervisees’ assessment of learning outcomes, and perceptions of the workshop	Skills F2F PS: didactic and active learning 10 h	Nursing Novice preceptors Teaching hospitals Taiwan	Gagné’s information processing theory (Gagné 1985)
McChesney and Euster (2000)	Mixed methods Quantitative scales and qualitative questions 22 educators Perceptions of the course and self-assessment of the learning outcomes	Knowledge and skills F2F PS: didactic and active learning 4 h	Social work Agency based social work field instructors Agencies USA	Knowles’ adult learning theory (Knowles 1972)
Methot et al. (1996)	Quantitative Longitudinal 1 manager, 4 supervisors, 7 direct care staff, and 16 clients Objective assessment of learning outcomes	Skills F2F PS: didactic, active and EL 3-h presentation and video viewing (duration of follow-up meeting not disclosed)	Mental health Supervisors A residential facility for persons with developmental disabilities UK	None
Murphy (2014)	Quantitative Survey 302 physical therapists Perceptions of the workshop and self-assessment of expected learning outcomes	Knowledge and skills F2F PS: EL 1 day	Physical therapy Clinical educators Workplace is not mentioned Canada	Benner’s novice-to-expert model (Benner 1984) Kolb’s experiential learning (Kolb 1984)
Quirk et al. (1998)	Quantitative Pre- and post-test, and follow-up 223 healthcare professionals Self- and objective-assessments of learning outcomes, and perceptions of the workshop	Knowledge and skills F2F PS: didactic, active and EL 1 day	Multiple professions: medicine, nursing, psychology and midwifery Clinician preceptors Workplaces not disclosed USA	None
Sandau et al. (2011); Sandau and Halm (2011)	Mixed Methods Quasi-experimental: pre- and post-test with qualitative comments 131 preceptors (experimental) and 74 (control group) Self-assessment of the learning outcomes, perceptions of the workshop, and supervisees’ perceptions of their orientation	Knowledge and skills F2F PS: didactic and active learning 8 h	Nursing Novice preceptors Hospital USA	None Novice-to-expert framework (Benner 1984) Adult learning theory (not specified)
Sayani et al. (2017)	Mixed methods Quantitative: pre- and post-test and qualitative interviews 50 midwives Objective assessment of learning outcomes, perceptions of mentoring and willingness to precept	Knowledge and skills F2F PS: not disclosed 2 days	Midwifery Novice preceptors Community Pakistan	None
Taylor et al. (2007)	Mixed methods Likert scale, free-text questions and focus groups 15 pharmacist preceptors Perceptions of the programme and self-assessment of learning outcomes	Knowledge Online PS: didactic and active learning 10–20 h	Pharmacology Rural pharmacy preceptors Community and hospital Australia	None

*F2F* face-to-face; *PS* pedagogical strategies; *EL* experimental learning; *h* hours

**Table 3:** Data extraction for extended-duration interventions

References	Study methods	Intervention	Settings	Middle-range theories
Halabi et al. (2012)	Qualitative Longitudinal design 12 nursing preceptors Self-assessment of learning outcomes	Knowledge and skills F2F PS: didactic, active and EL 1-week teaching phases with monthly 5-h meetings in between	Nursing Nursing preceptors Governmental and private hospitals Jordan	Kolb’s experiential learning (Kolb 1984) Dewey’s theoretical ideas of education as integration between theory, practice, reflection and action (Dewey 1964) Schön’s reflective practice (Schön 1987)
Milne and Westerman (2001)	Quantitative Observational and longitudinal design 1 consultant, 1 supervisor, and 3 supervisees Objective assessments of learning outcomes	Skills F2F PS: active and EL Hourly weekly meetings over an 8-month period	Mental health nursing Supervisor Community UK	Kolb’s experiential learning (Kolb 1984)
Myrick et al. (2011)	Qualitative 9/18 preceptors participated in semi-structured interviews Perceptions of the e-learning technology and self-assessment of learning outcomes	Skills Online PS: didactic and active learning 5 months	Nursing Preceptors 4th year of undergraduate nursing program Australia, Brazil, China, Hong Kong, Pakistan, UK and USA	Learning theory (non-specified) Experiential learning (non-specified)
Ögren et al. (2008)	Qualitative Longitudinal design 3 facilitators and 6 novice supervisors Self-assessment of group	Knowledge and skills F2F PS: didactic, active and EL 2 years (theoretical seminars 2-h weekly and group supervision 2-h weekly)	Psychotherapy Novice preceptors Workplace not disclosed Sweden	Psychodynamic theory (not specified)
Paulson and Casile (2014)	Mixed methods Pre- and post-test survey 40 nursing preceptors Self-assessments of learning outcomes and mental state	Knowledge and skills F2F PS: didactic and EL 1-day supervision workshop plus 6-month monthly follow-up peer group supervision training sessions	Rural mental health Rural supervisors Rural areas USA	None
Rogers and McDonald (1992)	Quantitative Quasi-experimental 25 field instructors (experimental) and 25 (control group) Objective assessment of learning outcomes	Knowledge and skills F2F PS: didactic, active and EL 20 h across 10 weeks	Social work Field instructors Workplace not disclosed Canada	Schön’s reflective practice (Schön 1987)
Seo and Engelhard (2014)	Mixed methods Quasi-experimental 21 physical therapist clinical instructors in experimental group and 24 in control group Self-assessment of learning outcomes	Knowledge and skills Online PS: active learning 9 weeks	Physical therapy Clinical instructors A public university USA	Knowles’ adult learning theory (Knowles 1990) Self-regulated learning (not specified)
Sevenhuysen et al. (2013)	Mixed methods Participatory research 14 clinical educators Self-assessment of learning outcomes and perceptions of the workshop	Skills F2F PS: didactic and active learning 2 h for each workshop (a series of 4 workshops with unclear timings in-between)	Physiotherapy Clinical educators Five hospital campuses and community health and rehabilitation centres Australia	Peer-assisted learning (not specified)
Sundin et al. (2008)	Quantitative Quasi-experimental 21 supervisors and 6 facilitators Self-assessment of learning outcomes and perceptions of supervisor styles	Knowledge and skills F2F PS: didactic and EL 2 years (group supervision is 2 h weekly: total 140 h duration)	Psychotherapy Novice supervisors Workplace not disclosed Sweden	Proctor and Inskipp’s theory (Proctor and Inskipp 2001)
Tebes et al. (2011)	Quantitative Quasi-experimental: pre- and post-intervention follow-up with no comparison group 81 social workers Perceptions of the training, self-assessment of learning outcomes	Knowledge and skills F2F PS: didactic and active learning 7-month duration (approximately 28 h or 5 days)	Social work Clinical supervisors Non-profit behavioural health agencies USA	Shulman’s interactional theory of clinical supervision (Shulman 1991, 1993, 2005)

### Short-duration supervision training interventions

Short-duration supervision training interventions typically focused on learning outcomes relating to supervisory knowledge and skills (content), were delivered face-to-face (mode) and employed multiple approaches such as didactic (e.g. presentations, videos), active (e.g. group discussions, case studies, reflection activities) and experiential learning (e.g. role plays, feedback). Although MRTs underpinning short interventions were often absent or not specified in the outputs, a range of theories were identified, the most common of which were adult learning theories (Knowles 1972), experiential learning (Kolb 1984) and the novice-to-expert model (Benner 1982).

Ten demi-regularities pertinent to our developing program theory were identified from the wide-ranging CMOCs identified in the extraction phase, with eight demi-regularities highlighting interventions’ positive outcomes and two demi-regularities illustrating interventions’ negative outcomes (see Table 4). In terms of the positive outcomes, all but one of the identified demi-regularities related to supervisor outcomes:

**Table 4:** CMOCs for short-duration interventions

Reference	CMOC	Illustrative quote (page number)
Busari et al. (2006)	Medical residents [C] who attended the 2-day teaching workshop [I] did not show significant improvement in teaching ability [− O] because of incomplete participation in the workshop [− M]	“The post-workshop ratings, however, showed no significant difference in teaching ability between the experimental group and the control group. Possible explanations for this finding could be the considerable drop-out… ill health, maternity leave, graduation, external clinical rotations and incomplete participation in the workshop.” (p. 140)
Carlson and Bengtsson (2015)	Participants [C] undergoing the CPD course [I] expressed their growth in self-confidence in relation to the preceptor role [+ O] through multiple learning activities [+ M]	“The participants explained, in interviews and reflective journals, how they had gained self-confidence in relation to the preceptor role through the different learning activities.” (p. 4)
	Preceptors [C] undergoing the CPD course [I] expressed that they would implement new educational models for students [+ O] due to newly acquired knowledge and skills [+ M]	“It was also described that the new knowledge and skills would be put to use to… implement new educational models for students.” (p. 4)
	Preceptors [C] undergoing the CPD course [I] improved precepting behaviours [+ O] as the preceptors had experienced an increased confidence in their abilities as preceptors [+ M]	“Reflective journals as well as discussions in the focus groups disclosed how participants experienced an increased trust in their abilities as preceptors. This was described as having gained inner strength and the courage to try new approaches to precepting.” (p. 4)
	Preceptors [C] undergoing the CPD course [I] improved preceptor behaviours [+ O] as they had gained new knowledge and improved communication skills [+ M]	“After participating in the course they [preceptors] expressed how the new knowledge and the improved communication skills helped them to be better prepared and courageous in situations they perceived as difficult.” (p. 4)
	Preceptors [C] undergoing the CPD course [I] shifted their didactical approach from teacher-oriented to learner-oriented [+ O] as they had gained more understanding of how they could use reflection [+ M]	“The preceptors perceived that they had gained more understanding of how they could use reflection as an educational tool working with the students. They explained that their didactical approach had shifted from teacher-oriented to learner-oriented.” (p. 5)
Clipper and Cherry (2015)	Nurse graduate supervisors [C] who participated in a preceptor-training program [I] may contribute to an improved transition to practice and improved first-year retention rates of NGRNs [+ O] due to structured preceptor-training program [+ M]	“A structured preceptor-training program may contribute to an improved transition to practice and improved first-year retention rates of NGRNs.” (p. 448)
Cox et al. (2017)	Given the various learning styles of each pharmacist preceptor [C], the online video mini-series [I] reached a broader audience [+ O] because the mini-series provide content in both written and video formats [+ M]	“Given the diversity of individual approaches to learning, we believe blended learning has the potential to more successfully reach a broader audience. Although this cannot be definitively said, the mini-series did provide content in both written and video formats.” (p. 9)
	Pharmacist preceptors [C] undergoing the online video mini-series [I] actively participated in reflection questions [+ O1] enabling them to apply the content to their preceptor role [+ O2] through the content relating to their experiences or hypothetical situations [+ M1]	“By requiring participants to think about a particular situation and how it would apply to them or their practice, they were able to process the educational content in a way that enabled them to more easily apply the content in their role as a preceptor.” (p. 10)
Eckstrom et al. (2006)	Internal medical preceptors [C] who participated in the 1-min preceptor (OMP) workshop [I] experienced increased use of the OMP teaching skills over the next 6 months [+ O] because their self-efficacy helped the continued performance of newly learned skills [+ M]	“Faculty who participated in our workshop felt that they increased their use of the OMP teaching skills over the next 6 months. Faculty perception of self-efficacy is critical to continued performance of newly learned skills.” (p. 412)
	Internal medical preceptors [C] who have participated in the 1-min preceptor (OMP) workshop [I] fell back to their previous patterns of supervisory behaviours [− O] if their new skills were not reinforced [− M]	“Because faculty are habituated to a particular teaching practice, they may make early changes after what they consider successful faculty development intervention, and then fall back into previous patterns of behaviour if the new skills are not reinforced.” (p. 413)
Ford et al. (2013)	Nursing and midwifery preceptors [C] participating in a 1-day workshop [I] increased their preceptorship confidence, knowledge and skills [+ O] based on the practice development approach [+ M]	“There is evidence of an interconnectedness between the development of knowledge and skills of the nurses and midwives and the enabling strategies that are utilised in the delivery of the program. Once again, these features of the program are also features of a Practice Development approach.” (p. 12)
Gillieatt et al. (2014)	Health professionals [C] participating in a 1-day training program [I] reported that their self-rated supervisory skills had changed as a result of the training [+ O] through engagement with practical exercises [+ M] together with feedback from peers and colleagues [+ M]	“The challenges associated with the simultaneous application of the three functions were explored through practice exercises using scenarios and through feedback from peers and trainers” (p. 4)
	Health professionals [C] participating in a 1-day training program [I] reported that their supervisory knowledge and skills had improved [+ O] through increased confidence [+ M]	“… reported that their skills had definitely changed (41%) or mostly changed (42%) post-training… feeling empowered, confident and enthusiastic; being more comfortable in the role of supervisor and having increased knowledge and skills.” (pp. 5–6)
Henderson et al. (2006)	RN preceptors [C] undergoing the 2-day preceptor training [I] increased knowledge about the preceptor role [+ O] due to the benefit of learning from others [+ M]	“One of the benefits perceived by the preceptors was their opportunity to learn from others. Some found that they learned from both new graduates and more experienced transfers.” (p. 133)
Hook and Lawson-Porter (2003)	Multiprofessional field work educators [C] involved in a 3-day fieldwork educator course [I] felt their educator and practitioner roles had changed positively [+ O] due to being more reflective on learning styles and supervision [+ M]	“…practitioners identified that their practice with students had changed positively, being more reflective with the use of learning styles and supervision skills. Some felt that they had changed in areas of practice within their role as a practitioner and not just within their educator role.” (p. 535)
	Multiprofessional field work educators [C] involved in a 3-day fieldwork educator course, plus reflective portfolio assignment [I] reported difficulty finishing assignment and course requirements [− O] because their service managers provided insufficient study time after the face-to-face session [− M]	“…most practitioners found the pressures of their workload and domestic life difficult to balance with the pressures of completing an assignment… not all participants were given study time to enable them to complete their portfolio.” (p. 535)
	Multiprofessional field work educators [C] involved in a 3-day fieldwork educator course [I] were satisfied with the course [+ O] due to the opportunity to share and discuss with other health professions [+ M]	“…the multi-professional and therapy-led nature of the course was well received. Participants welcomed the opportunity to share and integrate with other professionals and experience a programme that was relevant to their practice.” (p. 535)
	Multiprofessional field work educators [C] involved in a 3-day fieldwork educator course [I] were able to clarify ideas and consolidate work [+ O] because they engaged with their post-course mentor well [+ M]	“Some used their mentors in the early stages of the assignment to clarify ideas and help guide their thinking. Others used their mentors to consolidate their work and gain reassurance that their reflections were addressing the learning outcomes appropriately.” (p. 535)
Lee et al. (2017)	The nursing preceptors [C], after taking the training course including video instruction and reflections [I] experienced a strong pedagogical effect [+ O] because multiple teaching skills were presented in the course rather than using lectures alone [+ M]	“On the basis of the results of the focus group interviews with the NPs, the current authors thought that using nine instructional events… along with video instruction and reflections would have a strong pedagogical effect.” (pp. 226–227)
	The nursing preceptors [C] who participated in the training course [I] had their learning reinforced [+ O] after taking the course content-based quizzes [+ M]	“The reflection quizzes were all based on the content of the courses, reinforcing their influence.” (p. 227)
McChesney and Euster (2000)	Social work fieldwork instructors [C] involved in a 4-h workshop [I] felt the workshop had high productivity, involvement and safety [+ O] because of the use of active learning methods [+ M]	“Active learning methods were perceived to promote feelings of high productivity, high involvement and high safety for participation among field instructors.” (p. 201, abstract)
	Social work fieldwork instructors [C] involved in a 4-h workshop [I] participated in active discussion and effective interaction [+ O] due to brief lectures and discussion related to resource material [+ M]	“Brief lectures and discussion related to materials provided in the resource guide appeared to stimulate active discussion and effective interaction.” (p. 212)
	Social work fieldwork instructors [C] involved in a 4-h workshop [I] engaged in critical thinking and helpful discussion [+ O] due to prompts for reflective practice [+ M]	“Case vignettes and accompanying questions served to stimulate critical thinking and helpful discussion. The cases promoted discussion not only about specific practice dilemmas, but also about field instructor’s own experiences with similar situations and how they had been resolved.” (p. 213)
	Social work fieldwork instructors [C] involved in a 4-h workshop [I] were satisfied [+ O] and able to participate in constructive group discussion [+ O] due to the interactions amongst small group/small participant numbers [+ M]	“The small size of the seminar groups provided the opportunity for constructive group discussion. Most of the seminar participants actively engaged in discussion of field practicum issues.” (p. 213)
	Social work fieldwork instructors [C] involved in a 4-h workshop [I] were satisfied with and enjoyed discussion about a topic [+ O] due to the interaction with peers and exchange of views in a peer teaching activity [+ M]	“Each group read and discussed among themselves the ethical and legal dilemmas… Participants appeared to enjoy the interaction and exchange of views that this teaching method provided. They studied the article section assigned to them and lively discussion ensued.” (p. 213)
Methot et al. (1996)	Mental health supervisors [C] experiencing training videos demonstrating 10 components of a formal supervision meeting [I] achieved considerable behaviour change at the direct staff and client levels [+ O] as the supervisors and managers only needed to spend little time watching the videos to verify their on-the-job use of performance feedback skills [+ M]	“… it takes little time to verify on-the-job use of the performance feedback skills by trained supervisors. A great deal of behaviour change at staff and client levels can be achieved with a small amount of time invested in appropriate training at upper levels in the organization.” (p. 21)
	Mental health supervisors [C] experiencing training videos [I] showed variability in behaviour [− O] through the amount and types of other duties involved in managing, supervising and delivering direct care to developmentally disabled clients [− M]	“The variability in behavior levels for most subjects is apparent and should be qualified in terms of the amount and types of other duties involved in managing, supervising and delivering direct care… Because of these additional duties… one would not expect to find stable data on delivery of contingent consequences across observations.” (p. 21)
Murphy (2014))	Physical therapy clinical educators [C] participating in a hands-on workshop [I] were satisfied with the workshop [+ O] and had an increased perception of readiness and comfort to provide student learning [+ O] through role playing communication and conflict resolution [+ M]	“role play… [where] participants formed triads, with one person taking the student role, one the educator and one the observer… role-play was the most valuable thing learned.” (p. 338)
Quirk et al. (1998)	Community health preceptors [C] attending a 1 day face-to-face workshop [I] decreased the retention rate of teaching behaviours over time [− O] when complexity of initial learning increased [− M]	“Retention rates should decrease as complexity of the initial learning increases. In this regard one would expect the use of the teaching behaviours presented at the workshop might decrease over time more so than familiarity with the concepts.” (p. 707)
	Community health preceptors [C] undergoing a 1 day face-to-face short development workshop intervention using the education planning process [I] might maintain their appropriate use of teaching behaviours [+ O] because a booster session or incentive might reinforce learning and knowledge [+ M]	“Further studies are needed to see whether a booster session or incentives might help preceptors to maintain appropriate use of teaching behaviours… This would be especially important in situations where the faculty intervention is short and the expected change in behaviour is complex.” (p. 707)
	Preceptors from community health centres [C] attending a 1 day face-to-face workshop [I] can increase their familiarity with concepts [+ O] and their ability to use behaviours [+ O] as a result of a brief faculty development intervention [+ M]	“Community health centres can increase their familiarity with concepts and their ability to use behaviours a result of a brief inter-disciplinary faculty development intervention”. (p. 707)
Sandau et al. (2011)	Nurses from a large hospital [C] who participated in a mandatory 8-h preceptor workshop [I] had increased confidence, understanding and abilities in precepting new nurses [+ O] through workshop participation [+ M]	“Cohort 2 preceptors… At 3–6 months… Results for confidence and comfort in all five roles were significantly improved…” (p. 122)
	Nurses from a large hospital [C] who participated in a mandatory 8-h preceptor workshop [I] helped nursing orientees to increase their confidence on completing their first assignment [+ O] through supervisors completing training [+ M]	“… nurses in Cohort 2—orientees reported significantly greater confidence on completion of the first assignment after completing orientation compared with Cohort 1—orientees…” (p. 123)
	Nurses from a large hospital [C] who participated in a mandatory 8-h preceptor workshop [I] helped nursing orientees to increase their confidence in critical thinking [+ O] through supervisors completing training [+ M]	“… Cohort 2—orientees reported greater confidence in the use of critical thinking on completion of orientation… than did nurses new to the hospital.” (p. 123)
	Nurses from a large hospital [C] who participated in a mandatory 8-h preceptor workshop [I] helped nursing orientees’ retention within the hospital within a year of commencing their new role [+ O] through supervisors completing training [+ M]	“At 1 year post intervention, the proportion of new nurses (125 of 132) retained was significantly greater than in the previous year (82 of 94).” (p. 123)
Sandau and Halm (2011)	Nurses from a large hospital [C] who participated in a mandatory 8-h preceptor workshop [I] led to nursing orientees experiencing negative orientation [− O] because their preceptors did not have protected supervision time [− M]	“However, the quality of some preceptors was described as subpar, often because of lack of time.” (p. 176)
	Nursing preceptors [C] undergoing the workshop [I] had a negative precepting experience [− O] because of mismatched schedules of preceptors and orientees [− M]	“Mismatched schedules of preceptors and orientees, as a result of 8- versus 12-h shifts, rotating shifts, or preceptor vacation time, were cited as barriers to effective precepting.” (p. 179)
Sayani et al. (2017)	Midwifery participants [C] who have attended a 2-day mentorship workshop [I] did not begin to work as a mentor [− O] because they were occupied by other commitments [− M]	“The reasons for not beginning to work as a mentor were cited as busy work schedule, attending other training sessions, and personal commitments.” (p. 516)
Taylor et al. (2007)	The pharmacist preceptors [C], after participating in the online programme [I], experienced positive preceptor–preceptee relationships [+ O] as they changed their attitudes to their role of being a preceptor [+ M]	“Many participants experience a change in attitude to their role of being a preceptor and this indirectly affected his/her ability with the students.” (p. 51)
	The pharmacist preceptors [C] undergoing the online programme [I] can now access the training [+ O] as it is flexible in its delivery mode [+ M]	“All groups voiced their appreciation for the flexible delivery mode of the program which enabled them to access an educational course from their rural location.” (p. 50)
	The pharmacist preceptors [C] after undergoing the online programme [I] experienced confidence growth in their abilities to be an effective preceptor [+ O] as their prior experiential learning was validated, reinforced and extended [+ M]	“For many, their prior experiential learning was validated, reinforced and extended and lead to a subsequent increase in confidence in their abilities to be an effective preceptor.” (p. 51)

*NGRN* new graduate registered nurse; *RN* registered nurse; *NP* nursing preceptor

- Healthcare supervisors [C] undergoing short-duration supervision training [I] experienced improved satisfaction with training [+ O] (Hook and Lawson-Porter 2003; McChesney and Euster 2000; Murphy 2014); improved supervisory confidence [+ O] (Carlson and Bengtsson 2015; Ford et al. 2013; Taylor et al. 2007); improved supervisory engagement [+ O] (Cox and Araoz 2009; McChesney and Euster 2000; Taylor et al. 2007) and improved supervisory knowledge and practices [+ O] (C Cox et al. 2017; Ford et al. 2013; Gillieatt et al. 2014; Henderson et al. 2006; Hook and Lawson-Porter 2003; Lee et al. 2017; Methot et al. 1996; Murphy 2014) through mixed pedagogical strategies including active and/or experiential learning [+ M].
- Healthcare supervisors [C] undergoing short-duration supervision training [I] experienced improved supervisory practices [+ O] through improved knowledge, skills and/or attitudes [+ M] (Carlson and Bengtsson 2015; Taylor et al. 2007).
- Healthcare supervisors [C] undergoing short-duration supervision training [I] experienced improved supervisory practices [+ O] through improved confidence and/or self-efficacy [+ M] (Carlson and Bengtsson 2015; Eckstrom et al. 2006).
- Healthcare supervisors [C] undergoing short-duration supervision training [I] experienced improved supervisory satisfaction, knowledge, practices [+ O] through positive social relationships [+ M] (Gillieatt et al. 2014; Henderson et al. 2006; Hook and Lawson-Porter 2003; McChesney and Euster 2000).

The remaining demi-regularity relating to positive outcomes spoke to supervisee outcomes:

- Healthcare supervisors [C] undergoing short-duration supervision training [I] helped improve supervisee development and well-being (e.g. retention) [+ O] through structured training [+ M] (Clipper and Cherry 2015; Sandau et al. 2011).
  The demi-regularities that resulted in negative outcomes pertained only to supervisor-related outcomes:
- Healthcare supervisors [C] undergoing short-duration supervision training [I] experienced no improvements in supervisory skills [− O] through lack of engagement in training or reinforcement of training [− M] (Busari et al. 2006; Eckstrom et al. 2006; Quirk et al. 1998).
- Healthcare supervisors [C] undergoing short-duration supervision training [I] experienced poor supervisor engagement in training [− O] through insufficient protected time [− M] (Hook and Lawson-Porter 2003; Sandau and Halm 2011; Sayani et al. 2017).

**Fig. 3:**
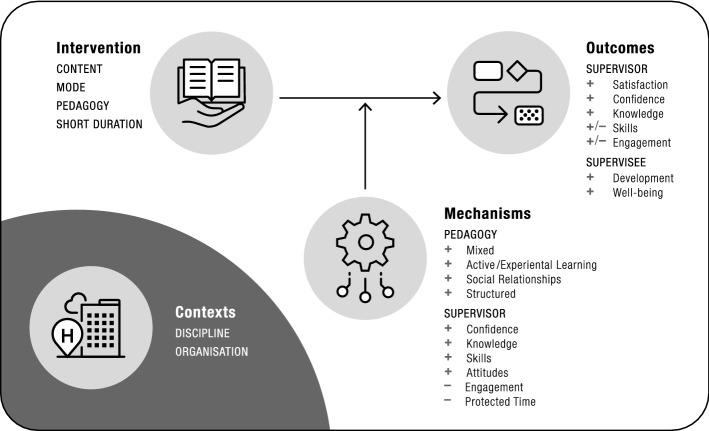
Modified program theory—short duration intervention

Based on these demi-regularities we developed a modified program theory (MPT) for short-duration interventions (Fig. 3).

### Extended-duration supervision training interventions

Extended-duration supervision training interventions also typically focused on participants developing their supervisory knowledge and skills (content), were delivered face-to-face (mode) and employed multiple approaches such as didactic (e.g. presentation, videos, reading), active (e.g. group discussions, reflective activities) and experiential learning (e.g. group supervision). Indeed, differences between short and extended-duration interventions (other than their longevity) were subtle, including: (1) some short-duration interventions being delivered online, and (2) more extended-duration interventions employing experiential pedagogical strategies. Although middle-range theories underpinning extended-duration interventions were sometimes absent or not specified in the outputs (e.g. ‘learning theory’, ‘psychodynamic theory’), various theories were identified. The most commonly identified were experiential learning (Kolb 1984), reflective practice (Schön 1987), and social learning theories (Proctor and Inskipp 2001; Shulman 1991, 1993, 2005).

We were able to identify fewer demi-regularities across our wide-ranging CMOCs for extend-duration interventions (Table 5). Firstly, we found five demi-regularities consistent with those already identified above for short-duration interventions but these were sometimes expressed in the reverse way (e.g. negative outcomes for extended but positive outcomes for short-duration interventions):

**Table 5:** CMOCs for extended-duration interventions

References	CMOC	Illustrative quote (page number)
Halabi et al. (2012)	Female registered nurses with more than 5 years’ clinical experience [C] undergoing a metropolitan university based part-time preceptor training program over 6 months (1 week every 3 months) [I] had improved their clinical teaching and capacity to guide students to integrate theory and practice [+ O] through the learning environment facilitating experiential learning pedagogies [+ M]	“The participants described how the pedagogical strategies improved their teaching in the practice area” (p. 139)
	Female registered nurses [C] undergoing a part-time preceptor training program over 6 months [I] had improved their ability to manage challenging learning situations [+ O] through the learning environment facilitating experiential learning pedagogies [+ M]	“In addition, most participants noted that they became better prepared to manage challenging learning situations while attending the program” (p. 140)
	Female registered nurses [C] undergoing a part-time preceptor training program over 6 months [I] had improved their communication skills [+ O] through the learning environment facilitating experiential learning pedagogies [+ M]	“Participants reported… improved communication skills with colleagues, students, and hospital staff” (p. 141)
	Female registered nurses [C] undergoing a part-time preceptor training program over 6 months [I] had improved student supervisees’ active involvement in group supervision sessions [+ O] through preceptors’ applying experiential learning pedagogies into group supervision [+ M]	“The participants stated that learning became easier and more fun when their students could share their ideas in a group discussion.” (p. 140)
Milne and Westerman (2001)	The supervisor [C] after undergoing the 8-month 1-h weekly consultancy and feedback [I] only received a modest outcome from the supervision activities [− O] because the consultancy was not sufficiently systematic [− M]	“Frisch (1989) used a carefully designed 40 h module to develop supervision skills… This included handouts and audio–visual aids, in conjunction with diverse teaching methods… Comparison with these two examples indicates, therefore, that the present study may have had its relatively limited impact because the consultancy was not sufficiently systematic.” (p. 454)
	The supervisor [C] after undergoing the 8-month 1-h weekly consultancy and feedback [I] only received a modest outcome from the supervision activities [− O] because the supervisees prefer amicable supervisor–supervisee relationships and exert a certain degree of influence over the supervisor–supervisee relationship [− M]	“… experiential learning methods are inherently challenging… effortful, and carry with them a considerable degree of uncertainty as to the outcome… This creates the conditions for the supervisees to counter-control or collude with the supervisor, as both parties would be more comfortable with the more supportive and non-threatening methods of learning…” (p. 454)
	The supervisor [C] after undergoing the 8-month 1-h weekly consultancy and feedback [I] only received a modest outcome from the supervision activities [− O] because the supervisor did not want to put too much pressure on already stressed supervisees [− M]	“… supervisees… experienced considerable stress in trying to implement the PSI [psychosocial intervention] methods… the supervisor often felt overwhelmed by the welter of stressors brought into supervision sessions… a collusive relationship was possibly shaped by negative reinforcement on both sides… an important threat to evidence-based supervision” (p. 455)
Myrick et al. (2011)	The nursing preceptors [C] undergoing the e-learning technology [I] were engaged to reflect on information [+ O1] and acquired precepting knowledge [+ O2] because a full-time facilitator interacted with them and ensured that they engaged in dialogue that was current, relevant, supportive and connected [+ M1]	“The social presence was ensured through the provision of a full time facilitator who engaged the participants in both synchronous and asynchronous discussion and interactive sessions throughout the trajectory of the program…” (p. 265)
	The nursing preceptors with lower computer literacy [C] undergoing the e-learning technology [I] acquired technological skills and ability [+ O] due to the opportunity to enhance their individual skill in the use of technology [+ M]	“The Virtual Space. [I]tself was found by the preceptors to be challenging and engaging while at the same time it was also found to provide them with an opportunity to enhance their individual skill in the use of technology.” (p. 265)
	The nursing preceptors [C] who received the e-learning technology [I] experienced interaction, learning and growth [+ O] due to the careful planning and integration of instructional strategies orchestrated by a competent instructional designer [+ M]	“In the planning of this online preceptorship support program, an instructional designer with a background in educational theory was consulted so that the appropriate learning tools could be adopted to facilitate an effective learning experience of the preceptors.” (p. 266)
Ögren et al. (2008)	When supervisors [C] undergoing the 2-year training program [I] were slow at understanding, the facilitators could help supervisors find their own pace of understanding [+ O] and alternative ways of seeing [+ O] when the facilitators were humble and patient [+ M]	“… supervisors believed that it was important that they assumed a humble attitude when they did not understand something that was expressed during the supervision. It was considered essential to wait for the supervisees and to let each of them find their own pace of understanding what was happening in the interplay.” (p. 12)
	The supervisors [C] undergoing the 2-year training program [I] had positive experiences of finding their paths to solutions [+ O] as the facilitators gave them space to reflect and think [+ M]	“The supervisees generally experienced that the program supervisors had actively sought to create space for the supervisees to reflect and ponder. Supervisees were given the opportunity to find their own paths to solutions” (p. 13)
	The supervisors [C] who underwent the 2-year training program [I] experienced security and quality in supervision [+ O] because the facilitator was direct and expressed themself clearly without being offensive [+ M]	“Something that contributed to security and quality in supervision was that the supervisor was direct and expressed him or herself clearly without being offensive” (p. 14)
	The supervisors [C] who underwent the 2-year training program [I] experienced an attitude of openness and curiosity when sharing their problems [+ O] through increased confidence [+ M] and feeling free to talk about their work without fear of judgement [+ M]	“This attitude [of openness and curiosity] was experienced as having contributed to an increased confidence… To feel free to talk about one’s work, to be able to associate with colleagues without being scared of being “right or wrong,” was emphasized as being important and a worthwhile aim.” (p. 13)
	The supervisors [C] undergoing the 2-year training program [I] could feel insecurity [− O] if the facilitators were extremely passive in style [− M]	“An extremely passive style could, however, create insecurity amongst the supervisees.” (p. 14)
	Supervisors [C] undergoing the 2-year group training program [I] improved their understanding that one situation could be managed in various ways [+ O] through listening and thinking about lots of perspectives on situations [+ M]	“Each group member is given the opportunity to follow the development of the other prospective supervisors and their respective therapists and clients over time. It becomes clear that one can manage similar situations in various ways depending on the particular circumstances.” (p. 15)
	The supervisors [C] in the 2-year training program [I] were led to new ideas and associations [+ O] through the interplay with other group members [+ M]	“Many supervisees reported that the interplay with the group members, which included giving attention to their presentations and receiving their views on their work, gave them new ideas and associations related to their own work” (p. 17)
	The supervisors [C] who underwent the 2-year training program [I] were struggling to find their place in the group [− O] as they were aware that each member’s time was limited [− M]	“Competition for time and a perceived lack of space contributed periodically to difficulties for supervisees in finding their place in the group” (p. 18)
	The supervisors [C] undergoing the 2-year training program [I] successfully shifted their perspective from being a psychotherapist to a supervisor [+ O] through the training program combining theoretical seminars and group supervision [+ M]	“Thus, this study provided support for the idea that supervision of prospective supervisees combined with theoretical seminars, in a unique way, contributes to this type of shift in perspective among supervisees” (p. 19)
Paulson and Casile (2014)	Rural mental health supervisors [C] undergoing a 1-day supervision training plus 6 monthly peer group supervision sessions [I] had decreased emotional exhaustion [+ O] and decreased depersonalisation [+ O] because they became more energized, connected and confident throughout the peer group supervision sessions [+ M]	“Overall, the group began without high levels of burnout and isolation, but still grew positively throughout the 6 months… the results suggest that the supervisors may have become more energised, connected, and confident throughout the peer supervision sessions” (p. 214)
Rogers and McDonald (1992)	Social work field instructors [C] undergoing a 10-week, 20-h program [I] had improved their ability to discriminate between truth and falsity [+ O] because of the pedagogical techniques employed facilitating supervisor reflection on inferences [+ M]	“This type of learning assignment, which forced them to actually reflect, consider, and articulate their inferences, might account for the increase in ability” (p. 174)
	Social work field instructors [C] undergoing a 10-week program [I] had improved critical thinking [+ O] through the learning environment facilitating critical reflection [+ M]	“The intent was to provide a learning environment and learning experiences that would facilitate and encourage critically reflective field instruction methods and practices” (p. 174)
	Social work field instructors [C] undergoing a 10-week program [I] showed no improvements in their abilities to determine if conclusions follow from information [− O] and no improvements in abilities to weigh evidence and decide whether conclusions are warranted [− O] because the course materials did not emphasise deduction or interpretation aspects of critical thinking [− M]	“There was little emphasis in the course in terms of content, exercise, or assignments on deduction, or on interpretation which may explain why there was no significant change in those areas” (p. 174)
Seo and Engelhard (2014)	Physical therapy clinical instructors [C] engaging in an online continuing education module [I] experienced perceptions of improved student mentoring quality [+ O] because of evoked motivation, critical thinking, self-directed learning and self-reflection [+ M]	“The online module evoked motivation, critical thinking, self-directed learning, and self-reflection and that the participants perceived an improvement in the quality of student mentoring.” (p. 49)
Sevenhuysen et al. (2013)	Physiotherapy clinical educators [C] undergoing four 2-h workshops to design and develop a peer-assisted learning (PAL) model of clinical education for paired undergraduate physiotherapy students [I] experienced improved engagement with the peer learning model [+ O] because facilitators adjusted the content of workshops, and model, based on feedback [+ M] and provided space during workshops to raise stakeholder concerns and develop solutions for concerns [+ M]	“… this level of engagement was achieved by responding to the continual critical review of stakeholder feedback and adjusting the content of the workshops, and the model itself, based on the feedback. It was also achieved by allowing “space” for participants to raise concerns and discuss potential solutions for these concerns” (p. 42)
	Physiotherapy clinical educators [C] undergoing four 2-h workshops [I] experienced improved engagement with the workshops [+ O] because of peer learning strategies used during the workshops as a strategy for engaging stakeholders [+ M]	“Peer assisted learning (educator to educator) was deliberately employed as a strategy for engaging participants in workshops, as clinical educators were encouraged to learn from one another’s experience and ideas” (p. 36)
Sundin et al. (2008)	Accredited psychotherapists (who had conducted > 125 psychotherapy sessions and practiced for > 3 years post-authorisation) [C] undergoing a 2-year part-time psychotherapy training program [I] gained knowledge and skills [+ O] through facilitators using more decisive styles [+ M]; and facilitators adopting authoritative approaches in the early to middle phases of the program [+ M]	“… a supervisor style that was perceived as more decisive (consultative, directive, active, structured) at 6 months contributed to perceived attainment of psychotherapeutic knowledge and skills at the 18-moth measurement” (p. 389).
	Accredited psychotherapists [C] undergoing a 2-year part-time psychotherapy training program [I] did not help trainees attain knowledge and skills [− O] if their supervisor prioritised a theoretical style [− M]	“… the negative association between self-ratings of a theoretical style and knowledge attainment could be taken to suggest that the trainees experience the task to integrate supervisory practice with theoretical considerations to be extremely challenging and frustrating” (p. 392)
	Accredited psychotherapists [C] undergoing a 2-year part-time psychotherapy training program [I] enhanced knowledge and skills [+ O] because of the positive relationships in a small group [+ M]	“The results suggested that the relationship among trainees was a substantial predictor of attained knowledge” (p. 391)
Tebes et al. (2011)	Licensed social workers with 16 years’ clinical experience and 8 years’ supervision experience [C] undergoing a 5-day interactional supervision training program over 7 months [I] experienced perceived increases in their competency [+ O] through participating in the training program and applying learnt skills in their supervision practice [+ M]	“… training in interactional supervision was associated with significant increases in supervisors’ perceived ability to manage supervisory relationships, manage supervisee job performance, and promote the professional development of their supervisees” (pp. 195–196)
	Licensed social workers [C] undergoing a 5-day training program over 7 months [I] experienced decreased supervisory stress [+ O] through understanding and applying approaches to managing the supervisory relationship [+ M]	“…managing supervisory relationships significantly predicts increases in supervisor stress management” (p. 195)
	Licensed social workers [C] undergoing a 5-day training program over 7 months [I] experienced increased satisfaction with their supervisory role [+ O] through understanding and applying approaches to managing the supervisory relationship and a decrease in supervisory stress [+ M]	“… increases in supervisor competencies are associated with increased supervisor satisfaction… managing supervisory relationships and managing job performance significantly predict increases in supervisor satisfaction” (p. 195)

- Healthcare supervisors [C] undergoing extended-duration supervision training [I] experienced improved supervisory knowledge and practices [+ O] through mixed pedagogical strategies emphasizing active and/or experiential learning [+ M] (Halabi et al. 2012; Myrick et al. 2011; Ögren et al. 2008; Rogers and McDonald 1992; Seo and Engelhard 2014).
- Healthcare supervisors [C] undergoing extended-duration supervision training [I] experienced modest outcomes only [− O] through a lack of systematic training involving mixed pedagogical strategies [− M] (Milne and Westerman 2001; Rogers and McDonald 1992).
- Healthcare supervisors [C] undergoing extended-duration supervision training [I] experienced improved supervisory knowledge and practices [+ O] through supervisor engagement [+ M] (Tebes et al. 2011).
- Healthcare supervisors [C] undergoing extended-duration supervision training [I] experienced poor supervisor engagement in training [− O] through insufficient protected time [− M] (Ögren et al. 2008).
- While healthcare supervisors [C] undergoing extended-duration supervision training [I] experienced improved supervisory satisfaction, knowledge, practices and/or attitudes [+ O] through positive social relationships [+ M] (Myrick et al. 2011; Ögren et al. 2008; Sundin et al. 2008), they experienced modest outcomes only [− O] through challenging social relationships [− M] (Milne and Westerman 2001; Ögren et al. 2008).
  We identified only one additional demi-regularity for the extended-duration interventions, not prominent for short-duration interventions:
- While healthcare supervisors [C] undergoing extended-duration supervision training [I] experienced improved supervisory engagement, knowledge and/or practices [+ O] through positive facilitator styles [+ M] (Myrick et al. 2011; Ögren et al. 2008; Sundin et al. 2008; Sevenhuysen et al. 2013), they experienced modest outcomes only [− O] through negative facilitator styles [− M] (Sundin et al. 2008).

**Fig. 4:**
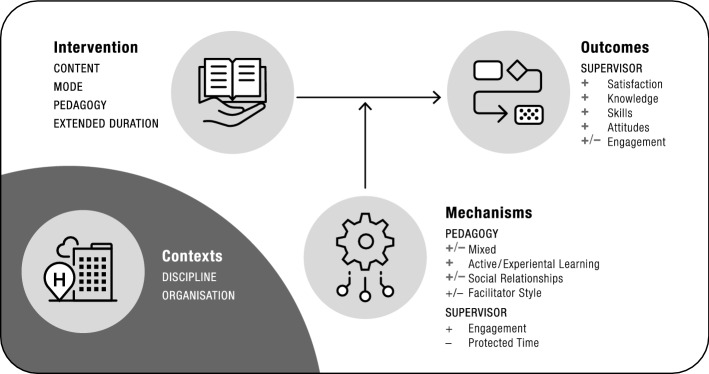
Modified program theory—extended duration intervention

Based on these demi-regularities we developed a MPT for extended-duration interventions (Fig. 4).

## Discussion

This synthesis set out to address the research questions: to what extent do supervision training interventions work (or not), for whom and in what circumstances, and why? Through our realist synthesis of 29 research outputs, we were able to develop two novel program theories, grounded in that evidence, about the positive and negative outcomes of short and extended-duration supervision training interventions, the mechanisms underpinning those outcomes and the extent to which those relationships were context-dependent, thereby developing existing knowledge on supervision training interventions.

### Summary of key findings

The developed program theories demonstrate that both short and extended-duration supervision training interventions have a multiplicity of positive supervisor outcomes including improved satisfaction, knowledge, skills, and engagement through a combination of mechanisms including mixed pedagogical approaches involving active and/or experiential learning, plus privileging social relationships (e.g. teacher–learner, peer–peer). Furthermore, both modified program theories illustrate that short and extended-duration supervision training interventions can lead to poor supervisor engagement in training when insufficient protected time exists for supervisor learning. Additionally, while most of the literature reviewed originated from health professions rather than human services contexts, we did not find that variations in disciplinary or organisational contexts were especially relevant to our program theories for short or extended-duration interventions. However, when comparing the mechanisms underpinning short and extended-duration training interventions, we found that *supervisor* characteristics (i.e. confidence, knowledge, skills and attitudes) were key mechanisms triggering positive outcomes for short-duration interventions, whereas *facilitator* characteristics were key mechanisms triggering either positive or negative outcomes for extended-duration interventions.

In summary, our findings are novel in two key ways: (1) that short *and* extended-duration interventions have numerous positive outcomes through mixed pedagogical approaches, social learning, and protected time for supervisors; and (2) that interventions of different durations may work in slightly different ways, with the success of short interventions relying on *supervisor* characteristics, and extended-duration interventions instead relying on *facilitator* characteristics.

### Comparison with existing literature

That mixed pedagogies involving active and/or experiential learning were important for the success of supervision training interventions is consistent with educational theories e.g. reflective practice (Schön 1987), experiential learning (Kolb 1984), plus our IPT based on three non-realist reviews of supervision training (Milne et al. 2011; Gonsalvez and Milne 2010; Tsutsumi 2011). Furthermore, that social relationships were also important for positive supervision training program outcomes in our modified program theories is also consistent with social learning theories (Shulman 1991, 1993, 2005; Proctor and Inskipp 2001). Finally, that negative outcomes occurred when supervisors were provided with insufficient protected time for learning, is consistent with literature emphasising the tensions between training and service delivery (Sholl et al. 2017). However, the findings of our realist synthesis not only extend our IPT but also add considerable new knowledge to the supervision training literature (Milne et al. 2011; Gonsalvez and Milne 2010; Tsutsumi 2011).

Firstly, our findings illustrate a wider range of outcomes (including negative outcomes) than has been previously identified in the supervision training literature including our IPT (Milne et al. 2011; Gonsalvez and Milne 2010; Tsutsumi 2011). Furthermore, aligned with our IPT based on previous non-realist reviews (Milne et al. 2011; Gonsalvez and Milne 2010; Tsutsumi 2011), we expected extended-duration interventions to have enhanced positive outcomes compared with short interventions, but we did not find this to be the case based on our realist synthesis of 29 outputs. It is worth noting that our synthesis included nineteen short and ten extended-duration studies from which to draw our conclusions, consistent with previous literature suggesting that short-duration supervision training interventions were more commonly delivered (Gonsalvez and Milne 2010). We did not identify additional positive outcomes for extended-duration interventions, plus we identified fewer demi-regularities across our identified CMOCs for extended-duration interventions. While this may reflect the fewer outputs reviewed in our study employing extended durations, our findings may reflect a genuine lack of added benefit from extending the duration of supervision training interventions. Indeed, healthcare workers may only require short interventions in order to realize key positive outcomes from training (as long as those short interventions include mixed pedagogies, social learning, protected time, and supervisor characteristics like confidence).

Secondly, our findings provide novel insights into the causal pathways for the multiplicity of ways in which both short and extended-duration supervision training interventions work (or not). Indeed, our realist lens has enabled us to identify the multiplicity of mechanisms triggered within supervision training interventions, leading to various positive supervisor outcomes. While interventions of any duration seemed to work through mixed pedagogies, social relationships and protected time (consistent with previous research and educational theories as described above), short interventions seemed to work through supervisor characteristics, whereas extended-duration interventions seemed to work (or not) based on facilitator characteristics. That learner characteristics seemed central in the face of short interventions mirrors previous research flagging the importance of supervisors’ personal qualities and skills as key contributors to supervision effectiveness (Wearne et al. 2012; Gibson et al. 2018), plus learning theories associated with the short-duration interventions, which were exclusively individualist and constructivist in nature such as adult learning theories (Knowles 1990), experiential learning (Kolb 1984) and the novice-to-expert model (Benner 1982). That extended-duration interventions seemed dependent on facilitator characteristics, probably relates to the increased importance of facilitator–supervisor relationships in the face of enduring associations (sometimes several years long). This also mirrors our finding that extended-duration interventions were associated with middle-range social educational theories.

### Methodological strengths and challenges

Our synthesis was strengthened through the use of a large multidisciplinary team and a rigorous process aligned with the RAMESES guidelines (Wong et al. 2013, 2017). However, we acknowledge several potential challenges concerning this synthesis. First, although we worked closely with a medical librarian and piloted our search terms, due to the voluminous nature of the supervision training literature, plus the extensive range of contexts included in our searches, we recognize that we inevitably omitted terms associated with supervision and/or training (e.g. coaching, facilitation etc.) (Lee et al. 2019). Therefore, we may not have identified *all* potentially key studies. Second, although our search strategy and inclusion criteria *did* include human services, this literature was either absent or excluded because of its poor quality and/or low realist relevance, meaning that our findings speak to health rather than human services contexts. Third, while we decided to include only peer-reviewed outputs due to the vast supervision training literature, we acknowledge that we may have excluded potentially important non-peer-reviewed grey literature, which may have been beneficial in the development of our program theories, and could have accounted for human services contexts. Fourth, none of the outputs included in our synthesis employed realist evaluation methods and as such, we struggled to tease out how context influenced the program theories. Fifth, like others who have identified a lack of evidence pertaining to the outcomes of supervision training on supervisees (e.g. students) and healthcare consumers (Gibson et al. 2018), the outcomes of studies included in our synthesis are somewhat limited to supervisor outcomes (and often based on self-report). Finally, the papers included in our realist synthesis often lacked explicit articulation of middle-range educational theories on which to base the development and refinement of our program theory. Furthermore, when theories were drawn on they were typically older individualist theories, rather than more sophisticated contemporary social educational theories.

### Implications for further research

Our study findings and our methodological challenges have a number of implications for further research. Firstly, given that our realist synthesis focuses primarily on health contexts, further literature reviews are now needed to explore supervision training in human services, perhaps employing different types of review (e.g. narrative review) to describe the types and outcomes of supervision training interventions for human services workers. Secondly, given that our realist synthesis has presented somewhat contradictory and unexpected findings about intervention duration, further research is now needed to explore more fully the similarities and differences between short and extended-duration supervision training interventions in terms of how they work (or do not work), for whom and under what circumstances, plus drawing on more contemporary social educational theories. The next stage of our supervision training study will employ realist evaluation (Wong et al. 2012), in order to explore the outcomes of short (i.e. half-day workshops) and extended-duration supervision training interventions for health *and* human services workers (i.e. half-day workshops plus 3-month longitudinal audio diaries), their underlying mechanisms and associated contextual nuances. Indeed, through employing realist evaluation we hope to better tease out how contextual variations influence mechanisms generating outcomes. Thirdly, similar to others reporting limitations in how the effectiveness of supervision training has traditionally been measured (Milne et al. 2011), further research is now needed that extends the ‘measurement’ of outcomes beyond supervisor outcomes to include outcomes for supervisees, and healthcare consumers. Indeed, realist evaluation could help to flesh out the multiplicity of outcomes for supervisors, supervisees and healthcare consumers, as well as identifying the multiple causal pathways.

### Implications for educational practice

Investment in supervision training has been proposed as having greater positive impact than resourcing supervision alone (Hill et al. 2014). In the quest to develop healthcare workers’ supervisory practices, we have found that supervisor training interventions of any duration can work to enhance supervisors’ confidence, knowledge, skills, and engagement through mixed pedagogical approaches involving active and/or experiential learning, privileging social relationships, and protected time. Supervision training that extends over longer periods of time showed no evidence of additional benefits in our realist synthesis. Our review therefore implies that only a modest investment may be required to produce significant outcomes for supervisory practices. These findings are important for resource-sensitive healthcare systems that fund the supervision training of healthcare workers. If offering short-term duration interventions, supervisor characteristics become important mechanisms triggering positive outcomes, whereas facilitator characteristics become central mechanisms triggering outcomes for extended-duration interventions. Therefore, we encourage healthcare educators involved in the design and facilitation of supervision training interventions to pay close attention to the key mechanisms highlighted in this realist synthesis in order to maximise the positive outcomes of supervision training interventions for supervisors. Finally, from an organizational perspective, supervision training programs need to be situated within organizational workplace cultures that enable supervisors to apply their new-found supervisory knowledge and skills to supervisory practices. Ultimately, healthcare organisations need to operate as positive learning organisations in order to maximise supervisory outcomes from training programs.
